# Surgical Treatment of Angular Pott’s Kyphosis with Posterior Approach, Pedicular Wedge Osteotomy and Canal Widening

**DOI:** 10.2174/1874325001711010274

**Published:** 2017-03-31

**Authors:** CV Kinkpe, M Onimus, L Sarr, MM Niane, MM Traore, M Daffe, AB Gueye

**Affiliations:** 1 Faculté de Médecine, Pharmacie et Odontologie de l’Université Cheikh Anta DIOP, Dakar, Sénégal; 2 8 chemin du cret F-25240 GELLIN, France; 3 UFR Santé de Thiès, Sénégal; 4 Centre Hospitalier de l’Ordre de Malte (CHOM), Dakar, Sénégal

**Keywords:** Pott’disease, Angular kyphosis, Pedicle substraction osteotomy, Vertebral resection, Neurologic risk, Posterior approach

## Abstract

**Background:**

It has been observed that the correction of severe posttuberculous angular kyphosis is still a challenge, mainly because of the neurologic risk.

**Methods:**

Nine patients were reviewed after surgery (mean follow-up 18 months). There were 2 thoracic, 4 thoraco-lumbar and 3 lumbar kyphosis. The mean age at surgery was 23.

Clinical results were evaluated by the Oswestry Disability Index (ODI) and by the neurologic evaluation. Preoperative, postoperative and final follow-up X-rays were assessed.

The surgery included a posterior approach with cord release and correction by transpedicular wedge osteotomy and widening of the spinal canal.

**Results:**

Average kyphotic angulation was 72° before surgery, 10° after surgery and 12° at follow-up. Three out of four patients with neural deficit showed improvement. Neurologic complications included a transitory quadriceps paralysis, likely by foraminal compression of the root.

**Conclusion:**

A posterior transpedicular wedge osteotomy allows a substantial correction of the kyphosis, more by deflexion than by elongation, with limited neurologic risks. However it is mandatory to widely enlarge the spinal canal on the levels adjacent to the osteotomy, in order to allow the dura to expand backwards.

## INTRODUCTION

The kyphotic spine deformity due to tuberculosis, described by Percival Pott in 1779, is nowadays infrequent in the developped countries, but vertebral tuberculosis is the main cause of severe angular kyphosis in the developing countries. The medical treatment is often delayed, and although providing satisfactory healing of the tuberculosis, there is a progression of the kyphosis until contact is obtained between the adjacent vertebral segments, with the creation of a bony block at the apex of the kyphosis [1].

Besides their psychological and social implications, angular kyphosis may result in severe cardiopulmonary insufficiency and moreover in early or late-onset paraplegia [2]. The deformity, the risk of progression and the risk of complications are arguments for surgery, as well as the occurrence of a neural compromise.

In this study, we reported the results of the surgery by posterior approach with transpedicular wedge osteotomy in the long-standing and rigid angular kyphosis.

## METHODS

This is a retrospective study. It included nine patients operated in between 2013 and 2015. No patient had previous orthopaedic or surgical treatment for kyphosis. Tuberculous disease was cured many years before and the general condition was good in all cases; the reason for referral in the patients was the spinal deformity and/or the onset of neural deficit. There were 2 thoracic, 4 thoraco-lumbar and 3 lumbar kyphosis (Table **1**).

There were five males and four females. The average age was 23 years (range 8-35).

Functionals results were evaluated using the ODI and by neurologic evaluation, recorded before the surgery, after the surgery and at the last follow-up. Radiographic measurements were done on preoperative, immediate postoperative and the last follow-up radiographs (in standing position). Due to the limited local facilities, no full spine standing x-rays were available and no measurements of the spinal sagittal balance could be performed. Mean follow-up was 32 months (range 20-44).

The patients were operated by posterior approach. The procedure included the initial insertion of pedicular screws in 2 or 3 vertebrae adjacent to the collapsed levels, then laminectomy was extended to one or two levels according to the desired correction. No temporary rod was used. After resection of the pedicles, both lateral aspects of the bony block at the apex were exposed and a wedge osteotomy was performed in the block. Chisel was used only to start the osteotomy and delimit the wedge resection; the main part of the resection was done with curette and disc forceps. The posterior cortex was kept in place during the procedure to protect the cord. It was removed at the ultimate stage. The osteotomy was extended close to the anterior cortex, but without reaching it. Then the spinal canal was carefully widened by chamfered excision of the anterior aspect of laminae and the capsulo-ligamentar tissues, in order to allow a posterior expansion of the dura during the correction of kyphosis. After introduction of the rods in the upper screws, a progressive and controlled correction was made by direct pressure on the lower part of the rods, until they could be introduced in the head of the lower screws, and by applying a slight compression between the upper and lower screws. The cord function was controlled by a wake-up test. Postoperatively early deambulation was allowed with the protection of a brace during 3 months.

## RESULTS

Average stay in the hospital was 15 days (10-30). Average operating time was 190 minutes (138-264). Average blood loss was 770 ml +/- 396. The ODI was 17 (1-29) before surgery (37,2%), and it was 6 (0-26) after surgery (12, 8%). Four patients had neurologic involvement: A spastic paraparesia (Frankel D) in a thoracic kyphosis did not improve ; a spastic paparesia (Frankel C) in a thoracic kyphosis fully recovered; two incomplete cauda equina syndroms in thoraco-lumbar kyphosis (Frankel D) fully recovered.

Preoperative average kyphotic angulation was 72° (55°-110°). Two vertebral bodies were collapsed in 6 cases (mean kyphosis 65°), three or four vertebral bodies in 3 cases (mean kyphosis 87°). The kyphosis averaged 85° at the thoracic spine (2 cases), 65° at the thoraco-lumbar spine (4 cases) and 71° at the lumbar spine (3 cases). Postoperative average angulation was 10° (-5° to 55°). It was 37° at the thoracic spine, 2° at the thoraco-lumbar level and 3° at the lumbar level. The angulation at the last follow-up was 12°. Neurologic complications included an unilateral quadriceps paralysis which fully recovered at 6 months, likely due to foraminal compression of the exiting root.

## DISCUSSION

The magnitude of the kyphosis is directly related to the number of destroyed vertebrae . Because of the destruction of vertebral bodies, kyphosis is sharp and angular, damaging the cord. According to Issack [3], surgery should be considered when kyphosis is more than 50°. However the severity of kyphosis is also related to the age of the patient when contracting tuberculosis. In childhood, the vertebral destruction is quite more accentuated and more progressive than in adults, where kyphosis is stable after healing [4]. In a study of 61 children who were under 15 years of age at the time of infection and who were followed during at least 15 years, Rajasekaran [5] observed that in 41% of cases, kyphosis continued to progress up to skeletal maturity after healing of the tuberculosis, and in more than 10% of cases progressed towards a severe angular hairpin type kyphosis (buckling collapse). The risk factors include age less than 7 years at the time of infection, thoraco-lumbar involvement, destruction of more than two vertebrae and « spine at risk signs » (subluxation of the facet joints at the apex of the kyphosis, posterior retropulsion of the affected vertebra, anterior toppling of the upper normal vertebra). According to Rajasekaran [6], in children a kyphosis of more than 30° and the presence of two « spine-at-risks signs» are arguments to consider surgery.

The correction of a severe deformity with sharp angular kyphosis requires an osteotomy of the three columns [7]. Yau [8] popularized a staged technique and progressive correction using halo-pelvic traction. This technique is nowadays no more used because of numerous inconveniences: moderate correction, increased risk of septic complications, difficulty of approaching the apex by transpleural thoracotomy, neurologic risk of a correction obtained more by elongation than by deflexion. More recent papers reported the association of anterior and/or posterior instrumentation, with simultaneous or successive sessions, the anterior approach being performed through a transpleural thoracotomy [9-12] or through an extrapleurel postero-lateral approach [13]; however these papers frequently concern active tuberculosis, which progresses slowly and is partially reducible.

**Fig. (1):**
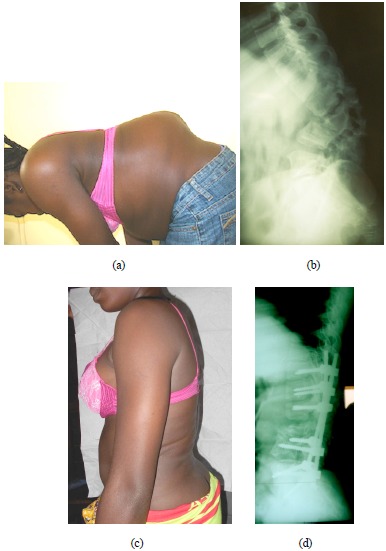
(**a-b**). Severe angular lumbar kyphosis involving destruction of L1, L2 and L3. Preoperative x-ray showing a 75° kyphosis. (**c-d**). Postoperative clinical and radiographic aspect after correction by pedicle substraction osteotomy and instrumentation from T11 to S1.

The correction of kyphosis by a single posterior approach with vertebral body resection is more effective and involves less neurologic risks, which is mainly done by deflexion than by elongation (Fig. **1**). The closing wedge posterior osteotomy was initially described for the correction of kyphosis in ankylosing spondylitis [14-16] and postraumatic deformities [17]. It was then used for the treatment of severe and rigid posttuberculous kyphosis [18, 19]. The procedure is basically the same; however the wedge resection is not carried out in a well defined vertebral body, but in the bony block resulting from several vertebral bodies’ collapse. This may explain why some authors mentioned a vertebral column resection with more or less complete resection of this bony block [20, 21].

**Fig. (2):**
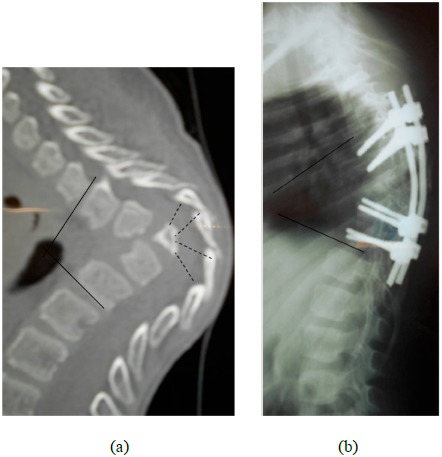
(**a**). Thoracic angular kyphosis with destruction of 4 vertebral bodies (the corresponding posterior arches can be seen). The patient presented a Frankel C paraparesia. (**b**). Same patient after correction with a T4-T11 fixation. The amount of correction was limited and the postoperative kyphosis is 55°. The patient improved to Frankel E after 3 months.

When two bertebral bodies were collapsed (6 cases), the average kyphosis was 65° ; it was corrected by a one-level laminectomy and one pedicle removal. When three or four vertebral bodies were collapsed (3 cases), the average kyphosis was 85° (Fig. **2**) ; it was corrected by a two-levels laminectomy and a wedge resection extended in the bony block as permitted by the laminectomies. In our experience, a complete vertebral resection of the anterior column, as advocated by some authors when three vertebrae or more are involved [22], is unnecessary. This is a more aggressive surgery with an extensive dissection and a total section of the contractured anterior fibrous tissues.

**Fig. (3):**
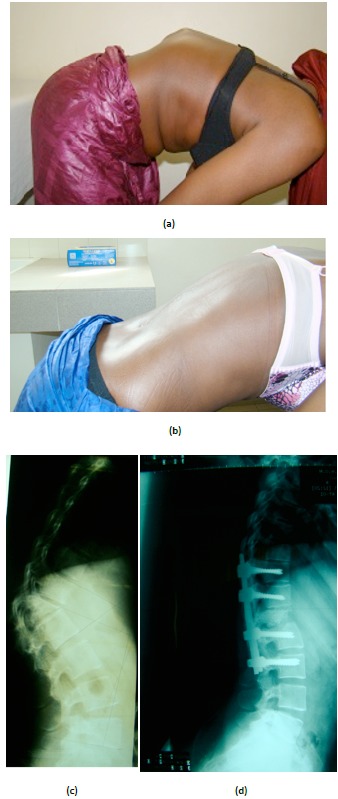
(**a-b**). Clinical pre et postopérative of patient N 4. (**c-d**). Xray pre et post operative of the same patient N 4.

At the thoracic spine, a complete correction of the kyphosis may not be beneficial because of the greater vulnerability of the spinal cord. In one case (Fig. **3**), the angulation was corrected from 105° to 55°; there was a subtotal paraplegia with complete incontinence which recovered completely at 3 months postoperatively. Furthermore an incomplete correction on the normally kyphotic thoracic spine may be better tolerated than a residual kyphosis on the normally straight thoraco-lumbar or lordotic lumbar spine.

In severe angular kyphosis, many authors have stressed [22-25] on the danger of an excessive shortening of the posterior column, and advised simultaneous opening of the anterior column, with the filling of the gap with graft or cage (the « closing-opening wedge osteotomy »). In an experimental study analysing the shortening of the cord in dogs, Kawahara [26] observed that a shortening of more than 12 mm buckled the dural sac, and the spinal cord kinked itself and was compressed by the buckled dura. However, the shortening was a purely axial one, therefore in a different situation from the shortening by the deflexion of kyphosis, where the shortening is not uniform and is observed on the posterior aspect of the dura, whereas the anterior aspect retains its length. According to Rajasekaran [1], in very severe kyphosis (hair-pin shaped), a longitudinal overgrowth of the horizontalised segments may occur with stretching of the cord at the apex, and at least theorically it should be preferable to shorten the anterior column than to lengthen it. On the other hand, it is mandatory to widely enlarge the posterior aspect of the spinal canal on both levels adjacent to the osteotomy, by chamfering the anterior side of the laminae and capsulo-ligamentar tissues, in order to allow the dura to conveniently expand and fold backwards.

Unlike several authors [20, 24], we did not use temporary rod during the vertebral body resection. As the resection did not include the anterior cortex, the spine was not destabilized, and moreover we never observed any spontaneous correction after wedge resection in spite of positionning the patient on thoracic and pelvic supports. The correction was obtained only after exerting correcting forces on the rods. The stiffness of the kyphosis may be not only due to the bony block, but also due to the thickening and contracture of the anterior longitudinal ligament.

As the monitoring of cord was not carried out in our institution, we examined the function of cord during the surgery by a wake-up test. In one case, although the patient actively moved her feet during the procedure, a postoperative complete unilateral paralysis of the quadriceps muscle was observed. The paralysis was regressive after six months. This radicular compression was likely due to an excessive narrowing of the foramen during the correction.

## CONCLUSION

Posterior closing wedge osteotomy is an effective way to correct severe post tuberculosis and angular kyphosis, giving a substantial treatment with minimal neurologic risks. The treatment was only partial on the thoracic spine and the canal was conveniently enlarged at the level of the osteotomy.

## Figures and Tables

**Table 1:** Clinical data.

N°	Age	Instrumented levels	Destroyed vertebrae	Preop angul	Postop angul	Last angul	Follow-up	Frankel	
								preop	F-Up
1	27	T10-L3	T12,L1	65°	-2°	4°	44 mths	E	E
2	18	L2-L5	L3,L4	63°	3°	4°	26 mths	E	E
3	25	T10-L3	T12,L1	55°	5°	5°	32 mths	E	E
4	22	T11-L3	L1,L2	68°	5°	6°	32 mths	D	E
5	35	T10-L3	T12,L1	72°	0°	4°	32 mths	E	E
6	19	T5-T10	T7,T8	65°	20°	20°	38 mths	D	D
7	35	T11-L5	L1,L2,L3	75°	-5°	-2°	38 mths	D	E
8	20	T10-S1	L1,L2,L3	75°	10°	12°	26 mths	E	E
9	8	T4-T11	T6,T7,T8,T9	105°	55°	58°	20 mths	C	E
